# Ethically challenging situations in eldercare: A cross-sectional study

**DOI:** 10.1177/09697330251317673

**Published:** 2025-02-06

**Authors:** Kirsikka Selander, Eveliina Korkiakangas, Risto Nikunlaakso, Tiina Koivisto, Jaana Laitinen

**Affiliations:** 3860 Finnish Institute of Occupational Health

**Keywords:** Ethically challenging situations, eldercare, employees, job strain, organizational constraints, organizational injustice

## Abstract

**Background:**

Ethically challenging situations are one of the many stressors that strain eldercare employees.

**Aim:**

The study aimed to examine (1) the mean levels of ethically challenging situations among eldercare employees in different Finnish eldercare service types and (2) the associations between organizational constraints and poor work-unit collaboration with ethically challenging situations.

**Research design:**

Cross-sectional survey in 2020 including 4,347 Finnish eldercare employees (response rate 67%). These employees provide care and support to older adults, such as assist with daily activities and manage medical needs. Employees were classified into four categories based on the eldercare service type: home care (*n* = 1,683), service housing (*n* = 1,649), outpatient and ward care (*n* = 650), and guidance and activity services (*n* = 365). The data was analyzed with variance analysis, t-tests, and linear regression analysis.

**Ethical considerations:**

The study was approved by the ethical board of the Finnish Institute of Occupational Health. Respondents’ provided informed consent for participation.

**Findings:**

Analyses showed that the level of ethically challenging situations was highest in service housing, among nurses, and among practical nurses. Organizational constraints—job strain and organizational injustice—had the strongest positive association with ethically challenging situations. Poor work-unit collaboration, instead, had a minor positive association with ethically challenging situations.

**Conclusions:**

Organizational constraints, especially job strain and organizational injustice, are important to identify to alleviate ethically challenging situations among eldercare workers.

## Introduction

The need for eldercare employees is increasing in the aging Western societies. The number of retiring eldercare workers is exceeding the number of new workers in the field.^1^ Simultaneously, high turnover rates are a serious problem in the health and social services.^2–5^ Thus, eldercare workers’ careers need to be sustained. Ethically challenging situations are one of the many job stressors that strain eldercare workers, being more prevalent in eldercare than elsewhere in health and social care.^6^ These situations are further associated with poor perceived work ability, especially when accumulating with other job stressors.^6^

Ethically challenging situations, where one knows what the right course of action is, but external obstacles prevent acting on it, evoke stress reactions that are referred to as moral distress in the nursing literature.^7–12^ Ethically challenging situations are usually found to derive from three main causes: (1) organizational constraints such as insufficient resources (lack of time and/or personnel and lack of institutional support); (2) patient-level factors such as causing unnecessary pain or suffering to patients and patient-family cooperation; and (3) work-unit level issues such as power imbalances among health care professionals and working with incompetent staff.^13,14,15,16,17^ Over time, stress caused by these situations can further lead to depression, increase sick leaves, and increase intentions to quit working.^7,13,14^

In this study we will focus on organizational constraints and work-unit collaboration issues that cause ethically challenging situations, as these factors can be more directly influenced by eldercare service employers than patient-related factors. In eldercare context, these constraints include organizational policies and leadership, decision making, resource allocation (time and staff), and competence or lack of social support.^18^ Reliable evidence of the causes and consequences of these situations among eldercare workers is, however, limited as most of the studies in eldercare have been descriptive with small sample sizes or using qualitative methods.^18^ Furthermore, to our knowledge, no quantitative analysis of ethically challenging situations in various Finnish eldercare service types has been conducted. Understanding causes of ethically challenging situations is essential in preventing moral distress caused by these situations.

Using a large dataset, the present study aimed to, first, explore the mean level of ethically challenging situations experienced by eldercare employees in different Finnish eldercare service types. The second aim was to examine the association between organizational constraints—organizational injustice and job strain—and ethically challenging situations, and the association between poor work-unit collaboration and ethically challenging situations. The findings of this study can help eldercare service employers to identify ethically challenging situations and structures and to plan tailored interventions to solve these situations at work in order to avoid moral distress.

## Data and methods

### Study design

This is a cross-sectional study of 4,347 Finnish eldercare employees.

### Data collection and participants

The study data were collected from an annual job well-being survey conducted in 9 Finnish public sector health and social care organizations. Participating organizations were selected based on their own interest. For this study, we used 2020 survey data. The employers provided the email addresses of all employees working actively during the time of the survey (from October to November). An email invitation to take part in an online survey and two reminders were sent to all employees. Of the invited employees (eligible population = 36,549), 24,459 responded (response rate 67%), and 92% gave consent to use their responses in scientific research (*n* = 22,502). A total of 4,347 employees working in eldercare work units were identified from the organizational structures provided by the employers and included in this study. Work units were divided into four categories often recognized in the Finnish eldercare system: home care (1,638 employees), service housing (1,649 employees), outpatient and ward care (650 employees), and guidance and activity services (365 employees).

In Finland, home care services include assistance with daily chores. They include support services such as meals, cleaning, safety and transport services, and health care and rehabilitation at home. Patients with a need for more support can receive a residence in an assisted living facility with 24-h care.^19,20^ Service housing includes a private room or apartment and a set of services based on an individual care plan. Short- and long-term institutional care (outpatient and ward care) can be provided for medical or safety reasons, but the use of ward care has decreased during the 2000s.^21^ Finnish eldercare services also include guidance and activity services targeted for older people and their formal and informal caretakers.^19^

### Measures

Ethically challenging situations were measured using a modified version of the scale created by Huhtala and colleagues.^22^ It contains two items: “how often do you have to act against rules and norms” and “how often do you have to act against your own values” (response scale 1 = never to 5 = daily). The overall score of the scale was obtained by a mean sum variable, high scores indicating more ethically challenging situations. The Cronbach’s alpha coefficient for the scale was 0.76.

### Organizational constraints

Procedural and relational injustice were measured with modified versions of scales developed by Elovainio and colleagues^23^ following Moorman’s approach to organizational injustice theory.^24^ Relational injustice consisted of four statements such as “My supervisor’s personal preferences do not interfere with his/her decisions” and “My supervisor treats his/her subordinates kindly and attentively.” Procedural injustice consisted of five statements such as “decisions made are consistent (the rules are the same for everyone)” and “effects of decisions are monitored and communicated.” The response scale was from 1 (totally agree) to 5 (totally disagree) in both measures. The overall score for both measurements was obtained as a mean sum variable, high scores indicating low organizational justice. Cronbach’s alpha for relational justice was 0.92, and for procedural justice, it was 0.93.

Measures for job strain were derived from the Job Content Questionnaire.^25^ Two items measured job demands, for example, “I am required to do an unreasonable amount of work,” and two items job resources, for example, “I have a lot of say in my own work” (response scale from 1 = strongly agree to 5 = strongly disagree). As recommended by Courvoisier and Perneger,^26^ job strain score was calculated by subtracting the mean of job resources from the mean of job demands. A higher score indicated higher job strain.

### Work-unit collaboration measures

Lack of social support from the work unit is a shortened version of the validated Team Climate Inventory.^27^ It includes items such as “we keep each other informed on work matters” and “everyone feels understood and accepted” (response scale 1 = totally agree to 5 = totally disagree). The overall score was formed as a mean sum variable, with high scores indicating a lack of social support from the work unit. Cronbach’s alpha for the scale was 0.89.

Inappropriate behavior was assessed using items from Statistics Finland’s Quality of Work Life Survey.^28^ It is a combination of two 1 = yes/0 = no questions—discrimination and bullying. For the analysis, employees who reported facing either or both of them during the previous 12 months were set as exposed to inappropriate behavior (1 = yes). Others were set as the reference group (0 = no).

### Background variables

As background variables, we used eldercare service type (home care, service housing, outpatient and ward care, guidance, and activity services), gender (male/female), age (years), supervisory position (yes/no), occupation, and perceived health. In Finnish eldercare, the largest occupation groups were practical nurses (69%) performing basic health care tasks, nurses (16%) with higher education and more complex duties, social counselors and social workers (4%) who assess social needs, provide counseling, and coordinate services, administration and clerical staff (3%), and personnel providing nutrition and/or cleaning services (2%). The remaining 6% were other health care professionals. Perceived health was considered poor if health was rated lower than good on a scale of good, fairly good, average, fairly poor, and poor.^29,30^

### Statistical methods

Descriptive statistics (means and percentages) were used to summarize participant characteristics. Group differences in ethically challenging situations were analyzed with variance analysis and pairwise comparisons with Fisher’s least significant difference *t* test (LSD). Associations between organizational constraints and ethically challenging situations, and between work-unit collaboration and ethically challenging situations were analyzed with Spearman’s correlation coefficients and linear regression analysis, controlling for gender, age, supervisory position, occupation, and perceived health. *p*-Values less than 0.05 were considered statistically significant. Data were analyzed using IBM SPSS Statistics 27.0 software.

### Ethical considerations

The study was approved by the ethical board of the Finnish Institute of Occupational Health. Respondents’ consent to participate in scientific research was requested in the questionnaire. The confidentiality and the anonymity of participants were guaranteed.

## Results

### Characteristics of the study population in different service types

Gender distribution and proportion of supervisors were similar in all service types (see Table 1). All service types were highly female-dominated (at least 94%) and comprised mostly employees without supervisory positions (at least 95%). Employees in home care were somewhat younger than in other service types. In both home care and service housing, 80% of the employees were practical nurses. They were the biggest occupational group also in outpatient and ward care (44%), where the number of nurses was also relatively high (37%). In guidance and activity services, the biggest occupational group was social counselors and social workers (30%). Poor health was most common in home care (33%). Correspondingly, in guidance and activity services, 8% of employees reported poor health.

**Table 1.** Participant’s characteristics.

	All (*N* = 4347)	Home care (*N* = 1683)	Service housing (*N* = 1649)	Outpatient and ward care (*N* = 650)	Guidance and activity services (*N* = 365)
Gender					
Male, *n* (%)	194 (4%)	98 (6%)	56 (3%)	27 (4%)	13 (4%)
Female, *n* (%)	4153 (96%)	1585 (94%)	1593 (97%)	623 (96%)	352 (96%)
Age, mean (95% CI), years	45.59 (45.23–45.94)	44.08 (43.50–44.67)	46.61 (47.19–47.12)	46.16 (45.25–47.08)	46.86 (45.78–47.94)
Supervisory position^a^					
Yes, *n* (%)	177 (4%)	59 (4%)	77 (5%)	29 (4%)	12 (3%)
No, *n* (%)	4156 (96%)	1618 (96%)	1568 (95%)	619 (96%)	351 (97%)
Occupation, *n* (%)					
Administration and clerical	119 (3%)	6 (0%)	17 (1%)	30 (5%)	66 (18%)
Nutrition and cleaning, *n* (%)	86 (2%)	20 (1%)	48 (3%)	18 (3%)	0 (0%)
Practical nurse, *n* (%)	3015 (69%)	1342 (80%)	1315 (80%)	284 (44%)	74 (20%)
Nurse, *n* (%)	689 (16%)	217 (13%)	194 (12%)	237 (37%)	41 (11%)
Social counselor and social worker, *n* (%)	173 (4%)	40 (2%)	10 (1%)	18 (3%)	105 (30%)
Others	265 (6%)	58 (3%)	65 (4%)	63 (10%)	79 (22%)
Poor health^a^					
Yes, *n* (%)	1327 (31%)	526 (31%)	542 (33%)	171 (26%)	88 (24%)
No, *n* (%)	3011 (69%)	1155 (69%)	1102 (67%)	477 (74%)	277 (76%)

^a^Supervisory position (*n* = 14) and poor health (*n* = 9) includes missing information.

### Ethically challenging situations in eldercare

On a scale of 1 to 5 (1 = never and 5 = daily), the mean level of ethically challenging situations in the study population was 2.78 (95% CI 2.75–2.81, see Table 2). It varied between service types, gender, supervisory position, occupations, and in relation to health status; between age groups, the group differences were statistically insignificant. The level of ethically challenging situations was higher among women (2.80, 95% CI 2.76–2.83), those not in supervisory positions (2.80, 95% CI 2.77–2.83), and those with poor perceived health (2.96, 95% CI 2.91–3.02). Contrarily, it was lower in guidance and activity services (2.40, 95% CI 2.29–2.51), differentiating from other service types (see pairwise comparisons in Table 3). Service and housing (2.86, 95% CI 2.81–2.91) differed also from home care (2.78, 95% CI 2.72–2.83), but not from outpatient and ward care (2.82, 95% CI 2.74–2.90). Most observed differences in mean levels of ethically challenging situations were, however, minor; they had only small explanatory power, explaining one percent or less of the total ethically challenging situations variation (see Table 2). Occupation had larger explanatory power, explaining three percent of the variance in ethically challenging situations.

**Table 2.** Mean levels of ethically challenging situations.

	Mean	95% CI
Eldercare service types (F = 18.42***), R^2^ = 0.00		
Home care	2.78	2.72–2.83
Service housing	2.86	2.81–2.91
Outpatient and ward care	2.82	2.74–2.90
Guidance and activity services	2.40	2.29–2.51
Gender (F = 15.91***), R^2^ = 0.00		
Male	2.48	2.33–2.63
Female	2.80	2.76–2.83
Age (F = 1.73), R^2^ = 0.00		
Less than 34 years	2.73	2.66–2.79
35–54 years	2.81	2.75–2.86
55 years or more	2.79	2.75–2.81
Supervisory position (F = 25.84***), R^2^ = 0.01		
Yes	2.38	2.26–2.51
No	2.80	2.77–2.83
Occupation (F = 25.24***), R^2^ = 0.03		
Administration and clerical	2.46	2.29–2.62
Nutrition and cleaning	2.00	1.76–2.24
Practical nurse	2.84	2.80–2.88
Nurse	2.92	2.84–2.99
Social counselor and social worker	2.59	2.45–2.73
Other health care professionals	2.14	1.98–2.31
Poor health (F = 55.41***), R^2^ = 0.01		
Poor	2.96	2.91–3.02
Good	2.70	2.66–2.74

Unadjusted means and 95% confidence intervals (CIs), F-tests of group differences, and explanatory powers (adj. R^2^).

***p-value <.001; **p-value <.01; *p-value <.05.

**Table 3.** Pairwise comparisons of ethically challenging situations across different service types. Table presents the mean levels of ethically challenging situations across different service types, including means and standard deviations (SDs). It also shows the unadjusted mean comparisons of ethically challenging situations between service types (mean difference and standard deviation) and the statistical significance (*p*-value) of the mean differences based on t-tests (*p*-values).

Ethically challenging situations	Home care	Service housing	Outpatient and ward care	Guidance and activity services
Home care	2.78 (SD = 0.03)			
Service housing	−0.08 (SD = 0.04)*	2.86 (SD = 0.03)		
Outpatient and ward care	−0.05 (SD = 0.05)	−0.04 (SD = 0.05)	2.82 (SD = 0.04)	
Guidance and activity services	0.37 (SD = 0.06)***	0.45 (SD = 0.06)***	0.42 (SD = 0.07)***	2.40 (SD = 0.06)

***p-value <.001; **p-value <.01; *p-value <.05.

Ethically challenging situations were highest among nurses (2.92, 95% CI 2.84–2.99) and practical nurses (2.84, 95% CI 2.80–2.88), followed by social counselors and social workers (2.59, 95% CI 2.45–2.73), and administration and clerical employees (2.46, 95% CI 2.29–2.62). The lowest levels of ethically challenging situations were observed among other health care professionals (2.14, 95% CI 1.98–2.31) and among nutrition and cleaning employees (2.00, 95% CI 1.76–2.24).

### Associations of organizational constraints and work-unit collaboration on ethically challenging situations

In a stepwise linear regression analysis, the first step introduced eldercare service types and ethically challenging situations as dependent variables. Employees in home care and in guidance and activity services faced ethically challenging situations less often than employees in the service housing (Table 4). These differences barely changed in the following steps.

**Table 4.** Associations between organizational constraints and work-unit collaboration with ethically challenging situations in linear regression analysis.

	Step 1	Step 2	Step 3
Service type (ref. = service housing)			
Home care	−0.04**	−0.04**	−0.04**
Outpatient and ward care	−0.02	−0.01	−0.02
Guidance and activity services	−0.12***	−0.09***	−0.07**
Gender (ref. = male)			
Female		−0.05***	−0.04**
Age		0.01	0.02
Supervisory position (ref. = no)			
Yes		−0.03	−0.02
Occupation (ref. = other health care professionals)			
Administration and clerical		0.04*	0.03
Nutrition and cleaning		−0.06***	−0.06***
Practical nurse		0.18***	0.10**
Nurse		0.18***	0.10**
Social counselor and social worker		0.07**	0.02
Poor health (ref. = good)		0.11***	0.03
Job strain			0.25***
Relational injustice			0.04**
Organizational injustice			0.15***
Lack of social support in the work unit			0.04**
Inappropriate behavior in the work unit			0.02
F change (df1, df2)	19.25 (3, 4241)***	21.20 (9,4232)***	130.82 (5,4227)***
Increase in the adj. R^2^	0.01	0.04	0.13
Adj. R^2^	0.01	0.05	0.18

Standardized regression coefficients and their statistical significance: ***p-value <.001; **p-value <.01; *p-value <.05.

Step two introduced background variables. Gender, occupation, and perceived health had a statistically significant association with ethically challenging situations; however, in step three, the differences in perceived health disappeared with some of the occupational differences. The association between occupation and ethically challenging situations disappeared for social counselors and social workers and for administrational and clerical workers in the third step. Among practical nurses and nurses, the association between occupation and ethically challenging situations decreased but remained statistically significant. Differences between male and female employees remained after step three.

After controlling for other variables, job strain had the strongest association with ethically challenging situations, with a regression coefficient of 0.25. Organizational injustice had a regression coefficient of 0.15 and for relational justice it was 0.04. Regarding work-unit collaboration, lack of social support from the work community had a weak positive association with ethically challenging situations (0.04). No main effect was detected on inappropriate behavior in the work unit. A separate correlation analysis found a statistically significant correlation between social support from the work community and inappropriate behavior (0.32, *p* < .001, Supplement Table 1), which may weaken the association between inappropriate behavior and ethically challenging situations. Of the total variance of ethically challenging situations, background variables explained four and organizational constraints with work-unit collaboration measures explained thirteen percentage points.

## Discussion

### Main findings

The present study showed, first, that the level of ethically challenging situations was relatively high in the service housing, outpatient and wardcare, and in home care. It was also high among female eldercare workers, those working as nurses and practical nurses, among workers without supervisory positions, and among those reporting poor health. The level of ethically challenging situations was, contrarily, lower in guidance and activity services than in other eldercare service types. Second, organizational constraints—job strain and organizational injustice—had the strongest positive association with ethically challenging situations. Poor work-unit collaboration had a minor positive association with ethically challenging situations.

To our knowledge, this is the first study analyzing associations between working conditions and ethically challenging situations in the eldercare context with a large sample size.^18^ However, our finding of the association between organizational factors and work-unit level issues on ethically challenging situations is consistent with previous research conducted on health care employees.^13,14^ Organizational factors, including job strain, however, had a stronger association with ethically challenging situations than work-unit level issues. Consistent with the finding of Rittenmayer and Huffman,^14^ ethically challenging situations were primarily associated with job strain, which limits employees’ ability to meet patients’ basic needs. The COVID-19 pandemic may have even exacerbated the effect of organizational constraints.^15,16^ Furthermore, this study found that work-unit collaboration had only a small association with ethically challenging situations. This finding aligns with a recent qualitative study by Glad and colleagues,^16^ who observed that team-level issues, such as feeling unsafe or bullied, were minor contributors, if any, to ethically challenging situations.

### Strengths and limitations

The main strength of this study is its novelty: it offers new information on the level of ethically challenging situations among eldercare workers and of the associations between working conditions and ethically challenging situations. Additionally, the study used a large dataset of eldercare employees, increasing the reliability of the findings.

The limitation of this study is, first, study design. Cross-sectional design prevents making conclusions about the temporality of the associations between organizational constraints and ethically challenging situations and between work-unit collaboration and ethically challenging situations. Thus, longitudinal studies are needed in the future. Second, the study lacked variables of the patient-level factors, for example, whether patient’s intensity of care could explain differences in ethically challenging situations between eldercare service types. Third, we used abbreviated measures of organizational constraints, which may oversimplify the measurements and reduce comparability with previous studies. Abbreviated scales can, on the other hand, decrease response burden, making them more feasible for participants and potentially increasing response rates. They have also shown to assess the same underlying concepts as the complete measurements.^31^ Fourth, work-unit collaboration variables used in this study measured inappropriate behavior and lack of social support from the work unit. They may not have captured the full scope of work-unit collaboration issues, including unequal power structures, discrimination, and unreliable colleagues which qualitative studies have identified as causes for ethically challenging situations.^14^ Fifth, this study was conducted in Finnish public sector eldercare organizations. The findings of this study may not be directly generalizable to eldercare organizations in other countries.

### Practical implications

The findings of this study emphasize the need for eldercare service organizations to recognize and tackle ethically challenging situations on the organizational level. Organizations need, first, to identify causes for job strain—high job demands and low job control—and how they cause ethically challenging situations. Reducing job strain in eldercare may be difficult, which emphasizes the importance of improving employees’ control over their work. Moreover, eldercare employers need to find ways to improve procedural and relational injustice. Improving the transparency and communication in decision making, and treating employees with respect and equality can make a difference. Ethically challenging situations could also be mitigated by lowering the organizational structure of the eldercare organizations and by supporting open discussion of ethically challenging situations inside the organizations. Improvements at the organizational level are likely to improve work-unit collaboration, which may explain why work-unit-level factors did not appear as strongly in the analysis as organizational factors. More research on this topic is required.

In our analyses, organizational constraints, work-unit collaboration, and background variables could only explain a small part of the total variance in ethically challenging situations. As the difference between service types remained statistically significant in the analysis, including patient- and family-related factors in the study could have improved the statistical power of the analyses. Employees in service housing constantly work in close contact with the same patients, more than the employees in other service types. Their work also includes close cooperation with relatives, and the patients require intensive care. Patients’ memory disorders can raise the issue of involuntary hospitalization and treatment, sometimes placing especially nurses in a difficult position between patients and doctors or relatives.^32^ Our finding of a relatively high association between working in service housing and ethically challenging situations may thus result from patient-related factors.

The findings of this study emphasize the need to consider ethically challenging situations also in different occupations, occupational levels, and among employees with poor health. The levels of ethically challenging situations were higher among nurses and practical nurses than in other health care professions. Especially nurses experiences of ethically challenging situations were high, which may originate from their role in eldercare organizations: they are responsible for organizing care and carrying out demanding nursing tasks,^21^ but they lack decision-making authority in comparison to physicians.^33^ Nurses and practical nurses work is straining, and working in a close contact with patients requires constant ethical consideration. Lower decision-making authority may also explain our observation of higher levels of ethically challenging situations among those in employee positions. The association between poor health and ethically challenging situations, in turn, may be related to the fact that as health deteriorates, an individual’s resources diminish, thereby reinforcing the feeling of strain. Furthermore, consistent with previous research,^7^ we found that women faced more ethically challenging situations than males. As in previous studies,^13^ differences across all sociodemographic variables were minor, and the underlying causes require further investigation.

### Implications for future research

This study has provided new information on the association between organizational constraints and ethically challenging situations and between work-unit collaboration and ethically challenging situations. To broaden the picture of the potential causes of ethically challenging situations, future studies should include patient- and family-related factors in the analysis. Furthermore, the instrument used in this study focused on ethically challenging situations but did not take into account the stress reactions they cause or the potential benefits, such as increased awareness of unethical behavior among caregivers. Therefore, more research is required on these topics. Finally, the findings of this study should be validated using a longitudinal data set and a different eldercare setting, for example, in a different country with a different cultural background.

## Conclusions

Ethically challenging situations evoke moral distress and are associated with employee retention. The findings of this study help eldercare employers to identify work units where ethically challenging situations must be addressed. The findings also provide guidance on how to approach ethically challenging situations. We found that the levels of ethically challenging situations were highest in service housing, among nurses, and among practical nurses. Organizational constraints—job strain and organizational injustice—had the strongest positive association with ethically challenging situations. Poor work-unit collaboration showed a minor positive association. While the study was conducted among Finnish eldercare workers, the findings of this cross-sectional study suggest that addressing organizational constraints and work-unit collaboration issues are important to identify and in alleviating ethically challenging situations. Although differences in health care systems between countries may influence the results, the findings are likely to be applicable, particularly in other Nordic countries. Furthermore, the findings can be used to tailor future intervention studies.

## Supplemental Material

Supplemental Material - Ethically challenging situations: A cross-sectional study Supplemental Material for Ethically challenging situations: A cross-sectional study by Kirsikka Selander, Eveliina Korkiakangas, Risto Nikunlaakso, Tiina Koivisto, and Jaana Laitinen in Journal of Nursing Ethics.
