# Supplementary material for: Ethically challenging situations in eldercare: A cross-sectional study
Source: Nurs Ethics. 2025 Feb 6;32(4):1035–46. doi: 10.1177/09697330251317673 (PMC12171036; doi:10.1177/09697330251317673)
Supplement: Supplemental Material - Ethically challenging situations: A cross-sectional study [file sj-pdf-1-nej-10.1177_09697330251317673.pdf]

Supplementary table 1. Correlation matrix. Pearson correlation coefficients and their statistical significance (p-values).

	Ethically challenging situations	Job strain	Relational injustice	Organizational injustice	Lack of social support from the work unit	Inappropriate behavior in the work unit
Ethically challenging situations	1					
Job strain	0.37***	1				
Relational injustice	0.22***	0.30***	1			
Organizational injustice	0.27***	0.31***	0.48***	1		
Lack of social support from the work unit	0.20***	0.29***	0.33***	0.38***	1	
Inappropriate behavior in the work unit	0.11***	0.14***	0.21***	0.17***	0.32***	1

*** p<0.001.
